# Recurrent Occipital Seizures with Transient MRI Changes

**DOI:** 10.1155/2017/6061879

**Published:** 2017-11-07

**Authors:** Mohankumar Kurukumbi, Allison Jacobs

**Affiliations:** ^1^Department of Neurology, Inova Fairfax Hospital, Falls Church, VA, USA; ^2^VCU School of Medicine, Inova Campus, Falls Church, VA, USA

## Abstract

Peri-ictal magnetic resonance imaging (MRI) findings following seizure activity are a recognized phenomenon that is not well understood (Cole, 2004). Transient changes are not usually expected to be present in postictal MRI studies because of their rarity. Here, we present a unique case of peri-ictal MRI findings located in the occipital lobe, present in a 34-year-old female with recurrent occipital seizures occurring twice in four years. MRI changes completely resolved after both episodes with no residual focal damage. The peri-ictal occipital changes on MRI in this patient are unique because they have been captured on more than one occasion. Peri-ictal MRI findings are a known phenomenon with unknown pathophysiology, although attempts have been made to understand these findings. Though the MRI findings and presentation appear to be stroke-like or PRES-like, seizures should be kept in the differential for better treatment outcomes.

## 1. Introduction

Peri-ictal magnetic resonance imaging (MRI) findings following seizure activity are a recognized phenomenon that is not well understood [1]. Although transient peri-ictal MRI findings are known to appear in some patients, they are reported anecdotally. Here, we present a unique case of recurrent peri-ictal MRI findings located in the occipital lobe, present in a 34-year-old female with recurrent occipital seizures occurring twice in four years.

## 2. Case Presentation

**Figure 1:**
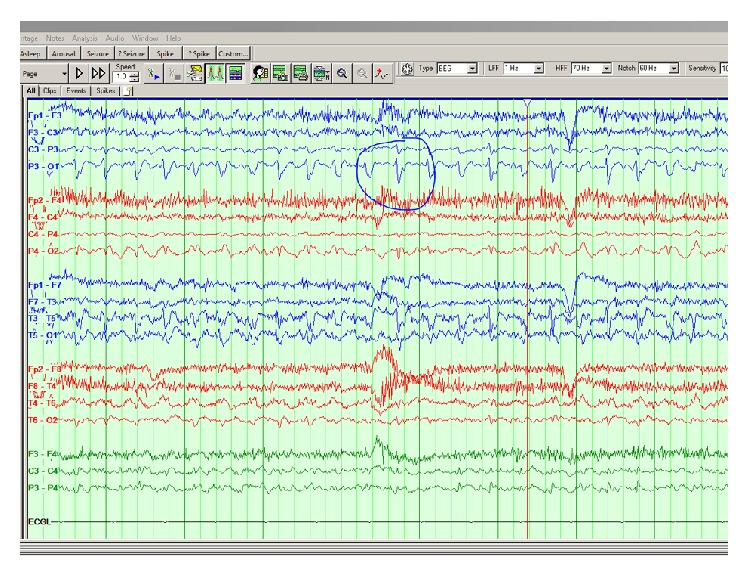
Bipolar EEG montage showing left occipital sharp waves (P3, O1) evolving into brief electrographic focal clinical seizures.

A 34-year-old female with a history of recurrent occipital seizures presented with a four-hour history of right visual field changes associated with left hemicranial headache. She described seeing a bright spinning beach ball in her right visual field intermittently, and physical exam showed constant right homonymous hemianopia. These symptoms were similar to those associated with a previous episode of clustered seizures that happened four years ago, at which point she was prescribed an antiepileptic agent, Levetiracetam. Continuous video EEG during the patient's most recent episodes showed left occipital sharp waves evolving into brief, frequent electrographic and clinical seizures, lasting 60–70 seconds (Figure 1).

**Figure 2:**
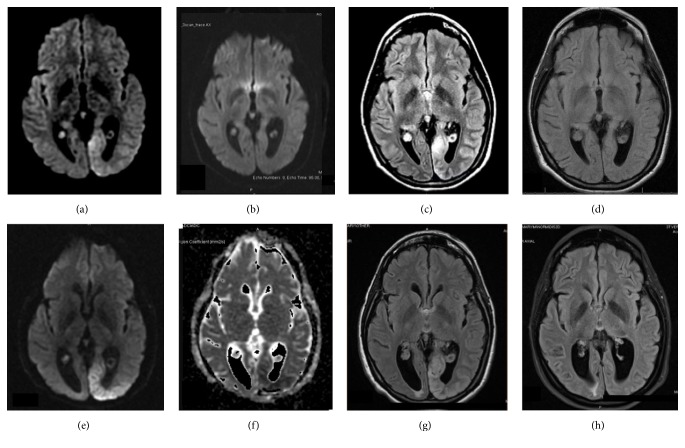
Axial brain MRI images of the patient showing transient left occipital enhancement following episode of clustered seizures in 2013 (a, b, c, d) and in 2017 (e, f, g, h). In 2013, (a) peri-ictal brain MRI-DWI shows diffusion restriction in left occipital cortex one day after her first reported seizure. (b) Follow-up MRI-DWI five weeks later shows complete resolution of abnormality in left occipital cortex. (c) Peri-ictal brain MRI T2/FLAIR shows hyperintense signal in left occipital cortex one day after her first reported seizure. (d) Follow-up MRI T2/FLAIR five weeks after seizure activity shows complete resolution of abnormality in left occipital cortex. In 2017, (e) peri-ictal brain MRI-DWI shows diffusion restriction in left occipital cortex one day after her first reported seizure. (f) Peri-ictal ADC map shows hypointensity in the left occipital cortex. (g) Peri-ictal brain MRI T2/FLAIR shows hyperintense signal in left occipital cortex. (h) Follow-up MRI T2/FLAIR six weeks following seizure activity shows complete resolution of abnormality in left occipital cortex.

MRI with DWI, ADC, and T2/FLAIR showed diffusion restriction and hyperintense signal throughout the left occipital cortex (Figures 2(e), 2(f), and 2(g)). Given this appearance, the primary diagnostic consideration was an infarct. However, there was no significant mass effect on the ventricular system, and asymmetric increase in vessels seen on the left side on the postcontrast imaging suggested luxury perfusion. There was no mass-like enhancement or edema to suggest an infectious or neoplastic etiology. The patient was successfully discharged with resolving symptoms after an additional antiepileptic agent was introduced. Follow-up MRI performed six weeks later showed that the lesions involving the left occipital lobe completely resolved without any residual encephalomalacia (Figure 2(h)).

MRI scans that were obtained four years ago showed increase in diffuse cortical and FLAIR signal abnormality involving the left occipital lobe with more focal diffusion restriction and hyperintense T2 signal medially (Figures 2(a) and 2(c)). EEG showed recurrent sharp wave activity along with focal slowing over the left occipital region, suggesting focal cortical irritability. Follow-up MRI five weeks later showed complete resolution (Figures 2(b), and 2(d)). She was placed on an antiepileptic medication at that time and had remained seizure-free for four years.

## 3. Discussion

Following seizure activity, MRI is routinely ordered to look for brain structural abnormalities. Nonstructural MRI changes are rarely seen in patients with epilepsy, especially in patients with focal seizures. Partial complex seizures associated with hippocampal involvement are reported in some case series [2], as well as irreversibility of MRI changes associated with status epilepticus. Recurrent unilateral reversible occipital peri-ictal T2-weighted hyperintensity, as seen in this case, is rare.

The pathophysiology to explain abnormal peri-ictal MRI findings is unclear at this point. It is believed that peri-ictal T2/FLAIR and DWI abnormalities that can sometimes be seen in patients with seizures reflect cytotoxic and vasogenic edema, induced by seizure activity in the brain [3]. Various theories are present in the literature regarding the cause of this edema. Horowitz et al. [4] attributed transient MRI enhancement to focally increased blood flow due to high metabolic rate at the epileptogenic focus, leading to increased oxygen consumption, hypoxia, hypercapnia, lactic acidosis, and vasodilation of cerebral vessels. In a report of temporary T2 signal hyperintensity in a patient with partial status epilepticus, Henry et al. [5] contrasted the pathophysiology of change in cortical white matter versus subcortical gray matter. They suggested that vasogenic edema was present in the white matter due to increased perfusion and permeability of vessels and that cytotoxic edema was present in the gray matter due to ischemia. Again, these relationships have not been pathologically proven.

At first glance, the brain MRI imaging suggests stroke, which may lead to stroke management erroneously. To confirm reversibility of the brain findings, follow-up MR imaging should be performed. Resolution of initial abnormal changes favors seizures, while, in stroke, residual encephalomalacia will be present. Additionally, it is important to consider the patient's clinical presentation and medical history, as well as the imaging results, before deciding on a treatment plan.

Another condition with similar findings on imaging is Posterior Reversible Encephalopathy Syndrome (PRES). On MRI, lesions appear as high signal intensity on T2-weighted images and FLAIR. The lesions are typically symmetric between the right and left hemispheres, although there is asymmetry in about 28% of cases [6]. On DWI, there is an increase in the diffusion coefficient. PRES can be confirmed if the reversible abnormalities on imaging occur in the setting of specific risk factors. These risk factors are arterial hypertension, eclampsia or preeclampsia, use of immunosuppressant medications or cancer chemotherapy, septicemia, chronic renal failure and dialysis, or autoimmune diseases, such as systemic lupus erythematosus, scleroderma, or Wegener's granulomatosis [7]. These factors were not present in our patient. The clinical manifestation of PRES can vary, but common symptoms include general seizures in combination with headaches, confusion, nausea, and vomiting. Focal neurological deficits may be present. It is important to recognize PRES because elimination of the inciting factor will lead to symptom resolution.

Evidence of recurrent peri-ictal MRI changes has rarely been reported [8]. There have been many reports of single episodes of MRI reversibility in patients, but cases of focal recurrent occipital seizures with local reversible MRI changes, as in this case, are not reported in the literature.

## 4. Conclusion

Transient changes are not usually expected to be present in postictal MRI studies because of their rarity. Therefore, we recommend follow-up MRI to confirm transient nature and to confirm that no permanent changes have occurred. No guidelines have been set regarding when to repeat imaging, but we would recommend approximately 60 days between initial and follow-up imaging. Larger studies are needed to determine the minimum time to expect reversibility of abnormalities on MRI, duration of antiseizure treatment in such rare cases, and a more detailed understanding of pathophysiology in these changes.

## Abbreviations

ADC: Apparent diffusion coefficient
CT: Computed tomography
DWI: Diffusion weighted imaging
EEG: Electroencephalogram
FLAIR: Fluid-attenuated inversion recovery
MRA: Magnetic resonance angiogram
MRI: Magnetic resonance imaging
PCA: Posterior cerebral artery
PRES: Posterior Reversible Encephalopathy Syndrome.
